# P02.160. The psychosomatic model for clinical oncology

**DOI:** 10.1186/1472-6882-12-S1-P216

**Published:** 2012-06-12

**Authors:** A Zhirkov, E Stepanchuk

**Affiliations:** 1 Saint Petersburg State University, Saint Petersburg, Russian Federation

## Purpose

The studies suggested a psychodiagnosis of cancer patients according to the SPb regenerative pyramid model.

## Methods

Thirty patients with lymphoma diseases, 30 patients with cancer of the reproductive organs (breast cancer and cervical cancer), and 39 patients with chronic myelogenous and lymphocytic leukemia were investigated.

## Results

The peculiarities of response to diseases in cancer patients were found according to the nosological group. It was shown that, despite the dominance of conventional adaptive ergopathic type of attitude to the disease (which is characterized by an obsessive attitude to work) in all studied groups, the presence of a high level of sensitivity component illustrates the lack of success to adapt to the situation of the disease. However, the presence of strong positive correlation between the type of response to disease and the level of depression in a group of hematological malignancies suggests a maladaptive system attitude to the disease in these patients. An analysis of coping behavior (by the method of Ways of Coping Checklist) of these categories of patients revealed a negative correlation between the level of depression and the most stressful coping strategies: seeking social support and positive reappraisal.

## Conclusion

The obtained results have theoretical value in understanding the regularities of formation of coping behavior of patients with cancer pathology in different nosological groups.

